# National recommendations of the Working Group for Post-analytics of the Croatian Society of Medical Biochemistry and Laboratory Medicine: implementation of autovalidation procedures

**DOI:** 10.11613/BM.2025.010503

**Published:** 2025-02-15

**Authors:** Vladimira Rimac, Jelena Vlašić Tanasković, Anja Jokić, Lorena Honović, Sonja Podolar, Jasna Leniček Krleža

**Affiliations:** 1 Department of Transfusion Medicine and Transplantation Biology, University Hospital Centre Zagreb, Zagreb, Croatia; 2 Department of Laboratory Diagnostics, General Hospital Pula, Pula, Croatia; 3 Department of Medical Biochemistry, Hematology and Coagulation, University Hospital for Infectious Diseases “Dr. Fran Mihaljević”, Zagreb, Croatia; 4 Medical Biochemistry Laboratory, General Hospital “Dr. Tomislav Bardek”, Koprivnica, Croatia; 5 Department of Laboratory Diagnostics, Children’s Hospital Zagreb, Zagreb, Croatia

**Keywords:** autovalidation, recommendations, postanalytical phase, clinical laboratory

## Abstract

Autovalidation is a computerised postanalytical tool that uses a sequence of procedures to verify laboratory test results without manual intervention. The Working Group for Post-analytics of the Croatian Society for Medical Biochemistry and Laboratory Medicine has prepared procedures for the implementation of autovalidation in routine laboratory work, which complement the existing national recommendations and aim to clarify the procedures of autovalidation. Before implementation, it is necessary to determine the need for the introduction of autovalidation in routine laboratory work, and then appoint the autovalidation team, whose task is to decide in which area of laboratory work autovalidation should be introduced, create the algorithm and supervise the verification of autovalidation. Standard rules included in the algorithm are patient data, messages from the analyzer, values of interference indices, autovalidation range and delta check. All criteria defined in the autovalidation algorithm have to be documented and approved by the laboratory manager. This autovalidation procedure shows the basic rules of autovalidation that can be used by any laboratory in the initial phase. The justification for using autovalidation will depend on the number and complexity of laboratory tests, the size of the laboratory personnel, and the available financial and material resources. Autovalidation avoids the subjective evaluation of laboratory test results as it is based on the same rules and is standardised to a certain extent, which further increases the quality of laboratory test results.

## Graphical abstract

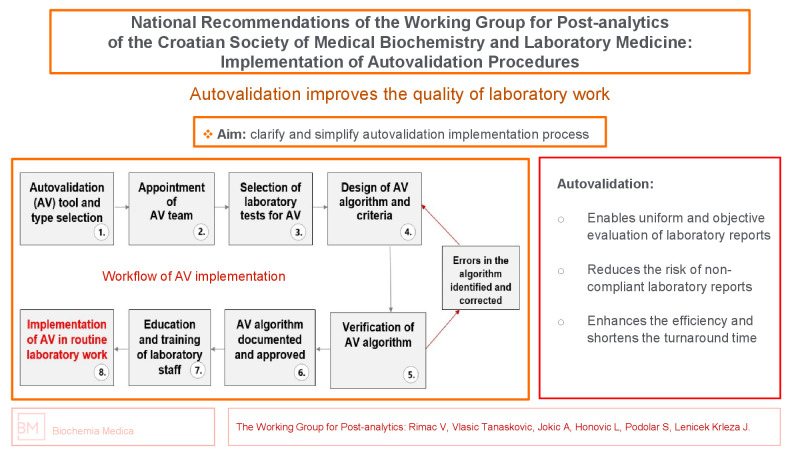

## Highlights

• Autovalidation is a computer-based postanalytical workflow tool

• Autovalidation shortens the time of laboratory test results release

• The autovalidation algorithm consists of a series of rules which each test result must follow

• The functionality of the autovalidation algorithm must be verified

• Laboratory test results that fail the autovalidation criteria must be reviewed and manually released

## Introduction

Automation of preanalytical and analytical phases has been present in medical biochemistry laboratories for many years, but it has also become an important part of postanalytical phase of laboratory work. Autovalidation is a computerised postanalytical workflow tool that uses a sequence of procedures to verify laboratory test results without manual intervention (*1*). The increasing number of requests for laboratory tests and the pressure to release reports in the shortest possible time emphasize the importance of autovalidation as a tool to improve the overall laboratory process, where achieving efficiency and increasing productivity are of great importance. By using autovalidation, it is possible to detect errors that occur in the preanalytical and postanalytical phases of laboratory work. All results are evaluated according to the same criteria, the time required to release numerous test results is reduced, and fewer laboratory experts are needed to review the laboratory test results before they are released (*2*, *3*).

Despite the relatively high level of computerisation of today’s laboratories in Croatia, there are still quite a few laboratories that have implemented autovalidation in their routine laboratory work (*4*). There are many reasons, such as insufficient personnel, the constant increase in routine work, but also fear of the new and lack of time to systematically read and study the literature.

To support laboratories at the national level in the implementation of autovalidation, the Working Group for Post-analytics of the Croatian Society for Medical Biochemistry and Laboratory Medicine (CSMBLM) has developed a procedure for the implementation of autovalidation in laboratory work. This procedure complements the existing national recommendations and aims to clarify and simplify the implementation process for autovalidation (*5*).

## 1. Preliminary actions for the implementation of autovalidation

**Recommendation:** Autovalidation software options should be chosen to ensure process efficiency with minimal complexity.Prior to implementation, it is necessary to determine the need for autovalidation and to assess the financial capabilities of the facility to support such a system upgrade.

Depending on the availability of such options, the rules and programming of the autovalidation algorithm can be embedded in the instrument software, laboratory information system (LIS), middleware, or any combination of the three. A key design objective is to choose the option that reduces complexity and enhances efficiency while still fulfilling the requirements for all tests involved in the autovalidation algorithm. Adjusting the autovalidation algorithm at various levels and combining programs can greatly enhance its productivity. However, the complex rules and synchronization of all calculation levels can be challenging during the initial setup and ongoing maintenance of the algorithm.

It is necessary to clarify with the LIS/middleware providers whether upgrading the existing system for autovalidation is possible. The capabilities of autovalidation software solutions and the process of releasing results must be understood before deciding on the most appropriate autovalidation tool. If automated validation is not possible with the current software, it is advisable to integrate a separate program for automated validation that connects to the LIS or middleware (*1*, *6*, *7*).

Furthermore, it is important to determine which type of autovalidation should be implemented: manually triggered autovalidation or real-time autovalidation. The first method requires the user to initiate the autovalidation process by clicking a button or icon, on the screen. The second method is real-time autovalidation, where test results are automatically validated immediately after the analysis is completed. If a laboratory initially adopts manual autovalidation, it can later upgrade to real-time autovalidation at any time. When real-time autovalidation is employed in routine laboratory work, it is essential to have a mechanism to stop the process at any moment. This precaution is necessary to prevent erroneous release of test results. After the initial agreements, the process of implementing the autovalidation system is initiated by the laboratory manager, who appoints the autovalidation team.

## 2. Appointment of the autovalidation team

**Recommendation:** The autovalidation team is responsible for the design, ver-ification, and implementation of the autovalidation algorithm into routine laboratory work.

It is recommended that the autovalidation team consist of laboratory professionals with a master’s degree in medical biochemistry and laboratory medicine, and those with a bachelor’s degree in laboratory diagnostics who are involved in routine work in the field of laboratory diagnostics for which autovalidation is planned. The person with a master’s degree in medical biochemistry and laboratory medicine is responsible for documenting all parts of the autovalidation implementation process and for appointing a person to enter the previously defined criteria into the system database through which autovalidation will be performed (*8*). The criteria set should be effective, simple, and user-friendly in detecting errors.

The members of the autovalidation team and their responsibilities should be documented and approved by the laboratory manager. The entire team participates in monitoring the system daily and takes corrective action when necessary. Another task of the head of the autovalidation team is to regularly inform the entire team and all employees about the status and improvements of the autovalidation system.

## 3. Creation of an autovalidation algorithm

### 3.1 Selection of laboratory tests for autovalidation

**Recommendation:** Autovalidation is primarily applied to automated tests that are most common in laboratories, such as routine clinical chemistry and hematology tests.

The first task for the team and the laboratory manager is to identify the area where autovalidation will be implemented. It is recommended that autovalidation is initially be applied to tests that are automated and most common in the laboratory, such as routine clinical chemistry or hematology tests. The number of tests, the turnaround time, the involvement of laboratory personnel and the time needed for manual verification, the number of administrative errors and other parameters of work quality - all these factors are crucial when assessing the need for the autovalidation of a test.

Regardless of the software used for autovalidation, the results entering the algorithm must be coming from regularly controlled analyzers where the reliable transfer of results from the analyzer to the LIS or middleware has been confirmed by previous procedures (*3*, *9*, *10*). If multiple analyzers are used in the laboratory, the autovalidation team can include or exclude certain analyzers from the autovalidation process.

### 3.2 Definition of rules in the autovalidation algorithm

**Recommendation:** Standard and ad-ditional rules and criteria have to be defined in the autovalidation algo-rithm.

**Figure 1:**
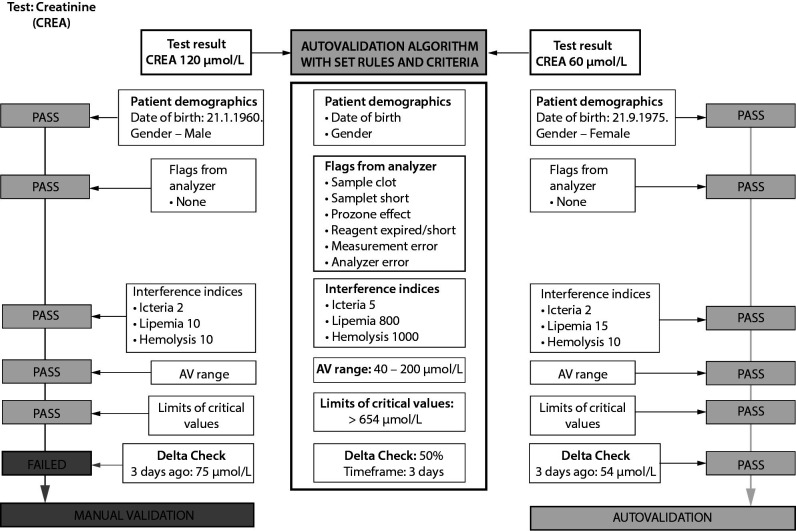
An example of an algorithm with defined rules for autovalidation.

The autovalidation algorithm consists of a set of rules that each test result must follow based on certain criteria. All rules in the algorithm are equivalent, and all test results must meet all the rule criteria to be autovalidated. Figure 1 contains a detailed example of an algorithm with defined rules for autovalidation. The number and complexity of the rules entered into the algorithm depend primarily on the capabilities of the software used for autovalidation, as well as on the laboratory’s standard operating procedures. Table 1 lists the standard and additional rules of the algorithm, regardless of the laboratory test for which autovalidation is used.

**Table 1:** Examples of standard and additional rules in the autovalidation algorithm

**Standard rules**	**Additional rules**
Patient demographics (date of birth, gender, location and/or physician)	Consistency checks
Messages from the analyser related to test results (instrument and data alarms/flags)	Quality control results
Sample integrity information (hemolysis, icterus and lipemia indices, clot)	Repeat testing
Limits of acceptance of test results (autovalidation range)	Reflex testing
Limits of critical values	Patient-based real-time quality control results
Delta check	Clinical diagnosis

#### 3.2.1 Standard rules

**Recommendation: **Standard rules to be included in the autovalidation algo-rithm are: patient demographics, mes-sages from the analyzers related to the test results, interference indices, auto-validation range, critical results, and delta check.

##### Patient data

The result is not autovalidated without the patient’s age and gender. This rule is important because results or reference values often depend on the patient’s gender and age. The age groups included in the autovalidation algorithm must be specified, as the laboratory may decide to exclude certain age groups from autovalidation (*e.g.*, neonates) (*9*, *11*). Other criteria related to the patient data, such as the diagnosis, the hospital department, and whether the patient is an inpatient or outpatient, may also be included in the autovalidation algorithm.

##### Messages from the analyzer related to test results

This rule includes all comments/messages from the analyzer that occur during the measurements and may influence the measurement results. These may include comments on sample quality (*e.g.*, sample clot, sample short, prozone effect), as well as messages relating to the analyzer itself (*e.g.* reagent expired/short, measurement error, analyzer error) (*10*, *11*).

##### Values of the interference indices

Interference index values (hemolysis, icterus and lipemia, HIL) should be set as a rule in the algorithm for all tests for which there is a (semi)quantitative automated determination of the HIL indices.). The laboratory is strongly recommended to introduce the measurement of HIL indices for routine clinical chemistry tests before implementing the autovalidation. However, if the laboratory does not measure interference indices, care must be taken to ensure that any preanalytical errors that may affect patient results are corrected prior to starting the autovalidation process (*e.g.*, such preanalytical interferences must be recorded, and new samples requested) (*11*, *12*).

##### Limits of acceptance of test results (autovalidation range)

The autovalidation range is defined for each test and implies a range within which each result passes through the autovalidation system. A result that falls outside the defined limits stops the autovalidation of the laboratory test result. Each laboratory sets its own limits for the autovalidation range, which may be, but are not limited to, the following: (a) analytical measurement range determined by method verification/validation, (b) limits of critical results, (c) selected threshold percentiles of cumulative patient results, (d) consensus of laboratory professionals, (e) reference intervals and (f) literature data (Table 2) (*3*, *12*, *13*). For example, historical results can be used to determine the 5th and 95th percentiles of all released results for a specific test. This method may reflect the local mindset and characteristics of laboratory patients in the most appropriate manner. In some cases, using limits based on clinically significant cutoffs (such as 11 mmol/L for glucose in the diagnosis of diabetes mellitus) or literature-based criteria may be the preferred approach. To achieve the best outcomes, it is important to use the limits established by most laboratory professionals reviewing the results. This approach ensures that the autovalidation will be tailored to local practices and well accepted by laboratory personnel.

**Table 2:** Different approaches for setting autovalidation and delta check limits in the autovalidation algorithm

**Autovalidation range**	**Delta check limits**
Reference interval	Reference change value
Reference interval limits minus/plus total allowable error	Percentile-based limits from the observed differences (*e.g.* 5th and 95th percentile in 3 days)
Midpoint between the median of the reference interval and critical results	Laboratory professional’s consensus
Critical results	Clinically significant change (*e.g.* troponin, creatinine, prostate-specific antigen)
Clinically significant limits	Literature data
Laboratory professional’s consensus	
Percentile-based limits from the cumulative patient results (*e.g.* 5th and 95th percentile)	
Analytical measurement range	
Literature data	

##### Limits of critical values

Critical values are laboratory test results that require urgent medical attention. If the test result is marked as critical, it should not be autovalidated but manually validated and immediately reported to the clinician.

##### Delta check

**Recommendation: **Delta check rule in the autovalidation algorithm should be set for tests that are frequently ordered and have a low index of individuality.

Differences between two consecutive measurements (delta check) can be the result of biological variability, changes in patient’s clinical condition, changes in therapy, but also errors in the preanalytical (*e.g.*, diluted sample, sample from the wrong patient, wrong tube type), analytical and postanalytical (transcription/transmission errors) phases of laboratory work (*14*). Advancements in laboratory medicine, including standardised protocols for sample labelling, the use of reliable and highly automated analyzers, automation in the total testing process, and modern LIS have significantly reduced the prevalence of such errors. However, sample misidentification and dilution by intravenous fluids still remains the most common preanalytical error detected by delta check. The main goal of this rule as part of the autovalidation algorithm is to recognise such errors while maintaining operational efficiency in the effort to address delta check violations (*15*-*17*).

Delta check should not be set for every test, but only for those that are frequently ordered and have a low index of individuality (low within-subject biological variation to between-subject biological variation). Generally, if the index of individuality of the measurand is below 0.6, the variability of results of an individual tends to stay within a narrow range compared to variability in a group of individuals. The expected accuracy of delta check in relationship to index of individuality has not been reported directly, but a strong correlation has been shown between within-subject biological variation and the performance of delta check (*18*, *19*). Table 3 shows the between-subject and within-subject biological variation of some measurands together with the accompanying index of individuality. Measurands like alkaline phosphatase, creatinine and mean corpuscular volume are better candidates for detecting misidentified samples than, for example, potassium and bilirubin, whose index of individuality is above 0.6 (*20*).

**Table 3:** Index of individuality, within-subject and between-subject coefficient of variation for some common biochemistry, coagulation and hematology tests

**Measurand**	**CVi (%)**	**CVg (%)**	**II**
APTT	2.8	7.2	0.39
ALT	11.4	35.2	0.32
AST	2.5	4.1	0.61
ALP	6.0	21.0	0.29
Bilirubin, total	20.2	24.6	0.82
Chloride	1.0	1.3	0.77
Cholesterol, total	5.2	15.3	0.34
Creatinine	4.4	16.2	0.27
RBC	2.8	7.0	0.40
Glucose	4.6	8.1	0.57
Hematocrit	2.8	5.6	0.50
Hemoglobin	2.7	6.2	0.44
MCV	0.8	3.9	0.21
Potassium	3.9	5.3	0.74
PSA	6.8	42.0	0.16
Protein, total	2.6	3.5	0.74
PT	2.6	5.1	0.51
Sodium	0.5	0.7	0.71
TSH	17.9	36.1	0.50
Urea	13.3	20.6	0.65
WBC	11.1	17.2	0.65
CVi - within-subject biological variation. CVg - between-subject biological variation. II - index of individuality. APTT - activated partial thromboplastin time. ALT - alanine aminotransferase. AST - aspartate aminotransferase. ALP - alkaline phosphatase. RBC - red blood cells. MCV - mean corpuscular volume. PSA - prostate specific antigen. PT - prothrombin time. TSH - thyroid stimulating hormone. WBC - white blood cells.			

The time interval for checking the difference between two consecutive measurements mostly depends on the patient population in the laboratory and the type of tests. In hospital laboratories, due to the expected greater change in test results and more frequent sampling for an individual patient, it is advised to use a shorter time interval (*e.g*., routine clinical chemistry tests 2-5 days), while in primary health care laboratories this interval can be significantly longer (*15*, *21*). There is no ideal delta check time interval, but the longer one is employed, the more likely it is that factors other than incorrect sample identification would account for the discrepancy in the test results. The shorter time intervals might also be preferred for practical reasons. When inspecting the delta check alarm, both the preceding and current sample should be investigated, which is usually limited to the archive capability of the laboratory and the stability of a measurand (*22*).

The next step in the procedure is to select the limits that will be used to indicate a delta check alert. The laboratory personnel’s experience, physician advice, or published literature can all be used to determine the limits. Alternatively, limits can be computed as percentile-based limits from the frequency distribution of observed differences in a chosen population, or they can be determined using data on biological variation and the calculated reference change value (RCV) of the measurand (Table 2) (*14*, *15*, *17*). Reference change value can be calculated using the data on within-individual biological variation from The European Federation of Clinical Chemistry and Laboratory Medicine (EFLM) Biological Variation Database laboratory analytical coefficient of variation for a specified measurand (*23*).

**Figure 2:**
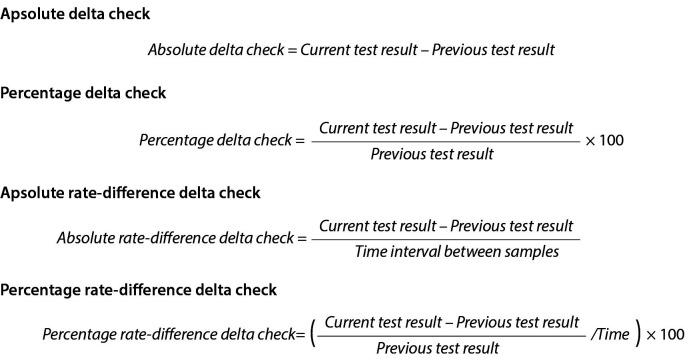
Common delta check calculations.

The most performed delta check calculations use either an absolute or a percentage difference over a period of time. For analytes whose concentrations are kept within strict limits, such as electrolytes, the use of absolute difference limits may be advantageous. However, for measurands such as enzymes where a larger change is predicted, percentage differences are recommended, especially in the high concentration ranges. The rate of change of the concentration of the measured value may be the preferred method for calculating the difference if such a change may indicate a significant clinical change (*e.g.*, the rate of change of creatinine to predict acute kidney injury, the rate of change of cardiac troponin in acute myocardial syndrome). Common delta check calculations are shown in Figure 2.

#### 3.2.2 Additional rules in autovalidation algorithm

##### Checking the consistency of the results

The rule of consistency of results applies to the results of two or more closely related tests, such as creatinine and urea, amylase and lipase, albumin and total protein. This rule is based on a predictable relationship between the results of each test and/or patient demographics and is used to detect analytical and preanalytical errors. Before entering the criteria for these results, it is necessary to define parameters that reflect these relationships in the LIS (*e.g*., computerised test for urea/creatinine, AST/ALT) and then set the acceptable limits (*3*, *24*, *25*).

Examples of consistency checks are: AST/ALT ratio < 0.25 or > 4; both TSH and fT4 less than lower reference limit, or above upper reference limit; urea/creatinine ratio outside 99.5% limits, albumin/total protein ratio < 0.25 or > 1, conjugated bilirubin/total bilirubin > 1, *etc*. However, several limitations affect predicted correlations, so it is important to keep that in mind when distinguishing between deviation due to the disease state, treatment outcomes and the effects of error (*11*, *26*).

##### Internal quality control (IQC)

It is recommended that this rule is used as part of the algorithm if a form of real-time autovalidation is used in the laboratory. The rule should be set to disable the autovalidation of test results if the internal quality control criteria are not met (*27*, *28*). In addition to the IQC rule, a time frame can be set after which the test results will no longer be autovalidated if the internal quality control does not meet the set criteria.

#### 3.2.3 Other rules that can be part of the autovalidation algorithm

In addition to the rules mentioned above, it is possible to define further rules, such as repeat tests, patient-based real time quality control, reflex testing. Some additional rules are outlined in Table 1, and laboratories will select which ones to incorporate into the algorithm based on their routine work. For example, if a patient-based real time quality control is used, it is recommended to include it in the autovalidation algorithm.

## 4. Verification of the autovalidation algorithm

**Recommendation: **The functionality of the autovalidation algorithm must be checked for all rules and criteria to ensure that patient results are not re-leased during the verification process.

Once the algorithm is defined with all rules and set criteria, verification of the algorithm must be performed (*28*). Before starting the verification process, all criteria for verifying the autovalidation must be defined and a predetermined percentage of autovalidated results should be met. During the verification process, all test results must be checked by all rules in the algorithm, and autovalidation must be stopped if any criterion in the algorithm is not met (*6*, *12*, *29*).

Before beginning the verification process, it is important to discuss the type of verification with the autovalidation software providers. If autovalidation is configured at the instrument or middleware level, it is crucial to ensure that the autovalidated results are not automatically released by the LIS. Autovalidated results should only be released by the LIS after a thorough check of the system’s full functionality.

On the other hand, if autovalidation is set at the LIS level, there are typically mechanisms in place to hold the results before their release. For example, an automated verification of the results may be initiated and recorded as autovalidation. In some cases, LIS providers can supply a test database to evaluate the algorithm used. In summary, any software designated as an autovalidation tool must include a reliable means of verification.

The criteria for verification procedures should be reasonable, *i.e.* a lower percentage of autovalidated results is expected when autovalidation is used in the laboratory for the first time. The goal of the verification procedure is to check the set rules in algorithm, but also to assess the need for a potential upgrade and improvement of the algorithm, such as the addition of a new rule or potential modification of a certain criterion.

Verification of autovalidation can be divided into two phases:

**Phase I:** The verification of the functionality of the defined algorithm (technical verification) by inclusion of simulated test cases so that all standard and additional rules be tested. Test cases can be manually entered into the LIS and/or middleware, autovalidation started (ensuring that no real patient results are released) and each tested result is noted with a ‘yes/no’ result for autovalidation. All boundaries of defined criteria must be verified by checking the values above, below and at the decision point itself for each test separately. Scenarios have to be simulated with the absurd and critical results, age and gender breaks, results with instrument flags and error messages, simulated test results with one result missing and cases with multiple rule challenges. Table 4 presents an example of a spreadsheet that can be used for the technical verification of autovalidation. Once the functionality of the autovalidation system has been tested for each test, the second phase of autovalidation begins.

**Table 4:** An example of a spreadsheet used for technical verification of the autovalidation algorithm set in the LIS for A) all tests and B) a single routine clinical chemistry test

**A) Institution:**					
**Department:**					
**LIS version:**		Sample ID* (Barcode)	**Result of AV (Yes/No)**	**Remark**	
**Result with expected NO outcome for AV**					
**AV rule**					
Patient gender absurd value					
Patient age absurd value					
Missing result for a test					
Instrument error affecting the result (code)**					
**Result with expected YES outcome for AV**					
**AV rule**					
Patient gender M or F					
Instrument error/message not affecting the result (code)**					
**Date:**		**Verifier name:**			
		**Verifier signature:**			
*Multiple samples may be checked. **Instrument error codes must be separately specified and verified. LIS - Laboratory Information System. AV - autovalidation. ID - sample identification within LIS. M - male. F - female.					
**B) Institution:**					
**Department:**					
**Test name:**		**Instrument:**			
**LIS version:**		Sample ID* (Barcode)	**Result of AV (Yes/No)**		**Remark**
**Result with expected YES outcome for AV**					
**AV rule**					
Age group included in the AV					
Data alarms and/or flags not affecting the result (code)**					
Result within:					
hemolysis index limits					
lipemia index limits					
icterus index limits					
AV range					
delta check limits					
Result out of delta check time interval (all other criteria met)					
Result at:					
low limit of AV range					
high limit of AV range					
Result included in additional rule within limits or followed by required actions (consistency check, repeat and reflex testing, PBRTQC)***					
**Result with expected NO outcome for AV**					
**AV rule**					
Age group not included in the AV					
Data alarms and/or flags affecting the result (code)**					
Result outside:					
hemolysis index limits					
lipemia index limits					
icterus index limits					
delta check limits					
Result below AV range					
Result above AV range					
Result included in additional rule outside limits or not followed by required actions (consistency check, repeat and reflex testing, PBRTQC)***					
**Date:**	**Verifier name:**		**Verifier signature:**		
*Multiple samples may be checked. **Data alarms and/or flags must be separately specified and verified. ***Each additional rule must be separately specified and verified. LIS - Laboratory Information System. AV - autovalidation. ID - sample identification within LIS. PBRTQC - Patient-Based Real Time Quality Control.					

**Phase II:** Manual validation and autovalidation are compared for each laboratory test result and noted in the corresponding form. It is important to note samples that have been autovalidated but not “manually” verified. Such samples require careful review by the autovalidation team and, if necessary, a change in the rules or criteria in the algorithm.

**Recommendation:** A comparison of autovalidation and manual validation must be performed. All possible errors and discrepancies must be carefully reviewed and corrected.

At the end of the verification process, the number (percentage) of autovalidated results is determined, and the manual validation and autovalidation are compared. Conclusions are also drawn about the verification of the autovalidation system.

## 5. Final procedures prior to the introduction of autovalidation in routine practice

**Recommendation:** The verification of the autovalidation algorithm must be documented and approved by labora-tory manager. Laboratory personnel need to be educated and trained before implementing autovalidation into rou-tine laboratory work.

All verification processes must be documented. The report must include information on the percentage of autovalidated results and the reasons for aborting the autovalidation if necessary. In addition, it is advisable to propose a procedure for validating the results that are not autovalidated (review of previous results, communication with the ward, request for a new sample). It is not necessary to include information on autovalidation of laboratory test results in a laboratory report. However, information about autovalidated laboratory test results must be available in the LIS or other software used for the autovalidation. In addition, the autovalidation team submits a report of the verification performed to the laboratory manager, who in turn approves the use of the autovalidation system in the laboratory. Before implementing autovalidation in routine work, laboratory personnel need to be educated and trained on the rules and criteria used in the algorithm.

In addition, the autovalidation team submits a verification report to the laboratory manager, who then approves the use of the autovalidation system in the laboratory. Before implementing autovalidation in routine work, laboratory personnel must be educated and trained on the rules and criteria used in the algorithm.

When there is a change in the algorithm or a change in the analytical part of laboratory work, the functionality of the autovalidation system should be checked. The functionality of the autovalidation system should initially also be reviewed regularly (at least once a year), and the established criteria modified if necessary (*11*, *30*).

## 6. Conclusion

The need to respond to the increasing number of requested laboratory tests and to produce and review them on time has led to the use of the autovalidation of laboratory test results in the daily work of medical biochemistry laboratories. This not only increases internal efficiency but also improves the quality of work. The use of autovalidation eliminated the subjective assessment of laboratory test results since as all results are evaluated based on the same criteria. It improves the quality of laboratory work by reducing the risk of non-compliant reports and minimizing the chances of delayed detection and reporting of critical results.

Given the variations in patient populations across different laboratories, it is essential to tailor the rules in the autovalidation algorithm to the specific needs of each laboratory. This includes taking into account the capabilities of the instruments, middleware, and LIS, depending on the tools used in the autovalidation process.

## Data Availability

All data generated and analyzed in the presented study are included in this published article.
